# Ter Die

**Published:** 1896-04

**Authors:** 


### "Ter Die."

It is not always good to be too curious, especially if you happen to be a hospital patient. One such was greatly concerned about what the physician wrote on a card at the top of his bed. While the nurse was not watching he took down the card, and immediately set up a great hullabaloo, groaning and sobbing in a dreadful manner. The nurse came to him asking him what was the matter. "Oh, dear! oh, dear!" was the response, "I've got to die!" "What is it? Do you feel worse?" asked the nurse in tender tones. "Not particular, mum, but I've got to die. The doctor has wrote it on my ticket." The poor fellow had so interpreted "ter die," and it was difficult to calm his fears. —*Exchange.*

